# Are They Really Trying to Save Their Buddy? The Anthropomorphism of Animal Epimeletic Behaviours

**DOI:** 10.3390/ani10122323

**Published:** 2020-12-07

**Authors:** Cédric Sueur, Marie-Amélie Forin-Wiart, Marie Pelé

**Affiliations:** 1 Université de Strasbourg, CNRS, IPHC UMR 7178, F-67000 Strasbourg, France; marie-amelie.forin-wiart@iphc.cnrs.fr; 2 Centre Européen d’Enseignement et de Recherche en Éthique, F-67000 Strasbourg, France; 3 Institut Universitaire de France, 75006 Paris, France; 4 Anthropo-Lab, ETHICS EA7446, Lille Catholic University, 59000 Lille, France; marie.pele@univ-catholille.fr

**Keywords:** empathy, comparative thanatology, cognitive biases, animal ethics, mentaphobia, primates, elephants, birds, robot

## Abstract

**Simple Summary:**

Anthropomorphism, defined as attributing human traits to animals and other entities, seems to have appeared during evolution to improve an individual’s understanding of other species (or indeed the world in general). Yet anthropomorphism can have beneficial or harmful consequences especially for animals, and there seems to be little interest in monitoring the potential danger of this approach. Few studies have focused on the factors affecting how we attribute intentions or beliefs to animals, and more quantitative studies are needed to identify how and why humans attribute mental states and cognitive abilities to other animals. In this study, participants answer questions about three videos in which an individual (a sparrow, an elephant and a macaque, respectively) displayed behaviours towards an inanimate conspecific that suddenly regained consciousness at the end of the footage. A fourth video showed a robot dog being kicked by an engineer to demonstrate its stability. These questions were designed to measure how far participants attribute humanlike intentions, beliefs or mental states to non-human animals and robots. Men and older participants are less likely to attribute humanlike mental states to animals. Similarly, people who work with animals or have at least one pet at home demonstrated less naïve anthropomorphism. Conversely, we found that members of animal protection associations showed more biophilia (affinity for other living organisms), attributed more intentions and mental states to animals and were further from biological reality (current scientific knowledge of each species) than non-members. Understanding the potential usefulness of these factors can lead to better relationships with animals and encourage human-robot interactions.

**Abstract:**

Anthropomorphism is a natural tendency in humans, but it is also influenced by many characteristics of the observer (the human) and the observed entity (here, the animal species). This study asked participants to complete an online questionnaire about three videos showing epimeletic behaviours in three animal species. In the videos, an individual (a sparrow, an elephant and a macaque, respectively) displayed behaviours towards an inanimate conspecific that suddenly regained consciousness at the end of the footage. A fourth video showed a robot dog being kicked by an engineer to demonstrate its stability. Each video was followed by a series of questions designed to evaluate the degree of anthropomorphism of participants, from mentaphobia (no attribution of intentions and beliefs, whatever the animal species) to full anthropomorphism (full attribution of intentions and beliefs by animals, to the same extent as in humans) and to measure how far the participants had correctly assessed each situation in terms of biological reality (current scientific knowledge of each species). There is a negative correlation (about 61%) between the mental states attributed to animals by humans and the real capability of animals. The heterogeneity of responses proved that humans display different forms of anthropomorphism, from rejecting all emotional or intentional states in animals to considering animals to show the same intentions as humans. However, the scores participants attributed to animals differed according to the species shown in the video and to human socio-demographic characteristics. Understanding the potential usefulness of these factors can lead to better relationships with animals and encourage a positive view of human-robot interactions. Indeed, reflective or critical anthropomorphism can increase our humanity.

## 1. Introduction

Despite their harmful effect on biodiversity, humans display biophilia [1,2], an innate affinity for other living organisms. More importantly, humans are interested in non-human animals [3]. This can be observed from the youngest age, when human babies are more attracted by animals than by inanimate or animate objects [4] and when their first vocalisations include many animal sounds [5]. While biophilia is possible in any individual, it is expressed in some people and cultures more than others [6]. During their evolutionary history, humans have also tended to attribute their own traits, emotions or intentions to non-human entities. This trend is well known—Darwin (1872) [7] noted that people tend to qualify non-humans as human-like beings—and is referred to as *anthropomorphism*. This term was first used in 1753, originally in reference to the heresy of applying a human form to the Christian God [8]. Although we may not always appreciate it or be aware of doing so, humans anthropomorphise not only animals but also objects or natural phenomena [9]. Anthropomorphism can have both beneficial and harmful consequences [10,11] for animals, and close attention should be paid to how we deal with it.

As far as human and animal relationships exist, the Animal (here, this term refers to non-human animals) has been conceptualised in three different ways: the humanised animal, the ‘beast machine’ and the animal as a sensitive being [12]. The humanised animal has been the most widespread conception in the history of civilisations, and consists of considering the animal as ‘a small or modified human’ [13]. Following this conception, animals have even been deified (as seen in Ancient Egypt) or put on trial in front of a court of justice, as seen in Europe in the Middle Ages (e.g., domestic cats, [14]). Much later, during the 17th century, it was good form to consider non-human animals as insensitive objects or machines, in line with Descartes and Malebranche’s postulate [15]. The use of the pronoun ‘it’ for an animal is a good example of objectification [16,17,18]. However, this mechanistic view of the animal was questioned in the following century by scholars such as Adam Smith, who stated in his Theory of Moral Sentiments (1759) that “*animals are not only the causes of pleasure and pain, but are also capable of feeling those sensations, they are far from being complete and perfect objects, either of gratitude or resentment* […]”. It is important to note, as shown by ethnographic records, that the cultural and religious backgrounds of humans influence their attitudes towards and interactions with other species [19]. For instance, monotheistic religions such as Judaism, Christianity and Islam are based on anthropocentric views and tend to argue that animals are ‘secondary creations’ designed to serve human interests, while vegetarian cultures such as Hinduism revere cows [20]. Recent research in animal behaviour and cognition provides clear evidence that animals are sentient beings (i.e., they have a capacity to feel, perceive or experience subjectively) that are capable of high cognitive capacities in the case of birds and mammals [21,22,23], although these capacities have yet to be proved in reptiles and fish [24,25,26,27]. We know that comparing human behavioural traits to those of animals, and particularly non-human primates, leads to a better understanding of the evolution and the development of these traits in humans [28,29,30,31]. However, it is important to recall that one century ago, using the terms ‘emotion’, ‘intelligence’ or ‘strategy’ in relation to animals was considered a scientific sin and an expression of weakness. Griffin subsequently created the term *mentaphobia* to describe the strong reluctance to refer to animal consciousness [32] when describing animal behaviour, as we cannot compare the incomparable [33]. This reluctance is also defined as *anthropodenial* [34].

Several different approaches were developed over the 20th century before an explicit definition of anthropomorphism was reached, in comparison to the automatic one [35,36]. Indeed, Norman’s theory of emotional design allows us to define three levels of anthropomorphism, namely visceral, behavioural and reflective [37] (or critical [38]) anthropomorphism. The visceral level is based on the first impression humans have of the physical attributes of animals. The behavioural level defines perception of animal behaviour and lastly the reflective level is based on how humans relate to the animal. Visceral and behavioural anthropomorphism are similar to anthropomorphism by omission, i.e., a failure to believe that other animals have a different world than ours, thinking that animals display the same capabilities and mental states as humans when showing a behaviour [39]. The case of the horse dubbed ‘Clever Hans’ illustrates how the hypotheses change from visceral/behavioural to reflective when observing the cognitive processes underlying the behaviours of this animal, which was known for its ability to resolve mathematical problems [40]: in fact, this horse was not a good mathematician but simply reacted to the emotions and facial expressions of the persons surrounding and watching him. According to Morgan’s canon [41], ‘in no case may we interpret an action as the outcome of the exercise of a higher psychic faculty, if it can be interpreted as the outcome of the exercise of one which stands lower in the psychological scale’ [42,43]. Thorndike [44] said that anecdotes [45] should be verified by behavioural experimentations that would logically be expected to reveal simple cognitive mechanisms in non-human animals. These philosophical and scientific Beast-Machine theories, also referred to as *mechanomorphism* [38,46], where, however, called into question by many studies showing that animals have complex emotions and cognitive capacities and may develop advanced and flexible social strategies [31,32,47,48]. However, it is possible that complex collective behaviours may also result from simple rules, contrary to the long-term belief of scientists that strong leadership and intelligence underpin such complexity in nest-building ants or flocks of birds [49,50,51].

In light of recent discoveries about cognitive complexity in animals (mainly in primates but see also findings in dolphins [52,53,54] or in parrots [48,55,56]), reflective anthropomorphism provided a better scientific hypothesis than Morgan’s canon: according to de Waal [29] and Griffin [32], this anthropomorphism was a more parsimonious theory than one that considered animals as unanimated objects or machines. However, anthropomorphism might also be an obstacle to the good understanding of animal behaviour and may lead to incorrect hypotheses and incorrect results in science, from both fundamental (do animals think?) and applied (do animals feel?) perspectives. Indeed, as *Homo sapiens*, we cannot think in any other way than as humans, and we are irredeemably destined to make judgment errors about animal sentience [29]. More generally, we may make suboptimal decisions due to well-known cognitive biases [57,58,59,60]. From an evolutionary point of view, the appearance of anthropomorphism (or ‘morphism’ if we are considering other species, e.g., *crotalomorphism* for snakes, *mamalomorphism* for mammals, or *macacomorphism* for macaques [39,61]) could be thought to have permitted individuals to gain a better understanding of other animal species and categorise them as prey, predator, competitor, informer, etc., thus increasing individual survival. This is relevant for some ethnic groups, which are primarily hunter-gatherers and animistic peoples (e.g., pygmy peoples or uncontacted tribes living in the Amazon rainforest). Many trackers and behavioural ecologists tend to shorten the distances between themselves and their model species, to improve their capture success for instance. It is also important to note all the rewilding processes observed in present-day societies [62]. Based on this hypothesis, anthropomorphism should not be arbitrary since the attribution of mental states to others is rooted in common and ancient brain substrates [63]. Cave art specialists state that “animals […] were integral to the evolution of the human brain to the extent that the encoding of animal forms seems to have become a dedicated domain of the visual cortex” [64]. This seems to be a logical evolution, as seeing and recognising animals (as species but also as individuals) was essential to survival.

However, whilst some individuals display anthropomorphism towards animals, others are more likely to express biophilia [6]. This variance, ranging from mentaphobia to constant anthropomorphism, is affected by different factors. Culture is a first factor affecting the way humans anthropomorphise. Following Descola’s postulates [65,66], the way we see or consider Nature (and animals as a part of it) is a social product which can follow four different modes of lectures or ontologies: animism (e.g., in Amazonia), totemism (e.g., in Aboriginal Australia), analogism (e.g., in India) and naturalism (solely in western cultures). How people empathise is linked to the way they anthropomorphise [67,68]. Empathy and anthropomorphism are two different concepts; empathy is the capacity to understand or feel what another human is experiencing. However the two concepts are linked insofar that they are managed by the same parts of the brain: the posterior superior temporal sulcus [69] and the mirror neurons [70] react to the facial expressions of humans [71], animals or robots [72,73]. Diet provides a good example of the link between the two concepts: vegetarians show a higher brain response of empathy-related areas than omnivores when observing scenes of animals suffering [74]. The scientific knowledge possessed is another factor affecting the likelihood of committing fewer errors when anthropomorphising. In this way, attributing anthropomorphism to human perceptions is subject of discussion among scientists as one may suggest that if some species have similar characteristics to humans because of sentience, advanced cognitive skills, etc., then human perceptions of those characteristics or behaviours are not anthropomorphic. However, human perceptions are personal and as a consequence, anthropomorphism is personal. This means that, even if the cognitive capacities of a species is well established among the scientific communities, a person who does not possess this knowledge will make anthropomorphism when observing this species. This is exactly the anthropomorphism by omission. However, the expert scientist does not make any more anthropomorphism when observing this species. A good example of this is tourists visiting the Atlas Mountains in Morocco or Algeria and facing Barbary macaques (*Macaca sylvanus*). As non-experts, they mistakenly read and interpret the ‘bared-teeth’ facial mimic of these monkeys as smiles when they are actually real threats that may end in serious conflicts with the animals. However, such errors of judgment may easily decrease with experience but also with educational boards providing correct information to visitors [75]. In the case of pets, dogs behaviours are also well and correctly interpreted by people who have experience with them [76,77]. However, dog owners may be more likely than professionals to wrongly attribute intentions to their pets [78,79]. The complex social behaviours of cats are frequently misunderstood by owners, jeopardising their species-specific needs and impacting their welfare [80]. Few studies focus on the factors affecting anthropomorphism, and more quantitative studies are needed to identify the factors influence how humans attribute mental states and cognitive abilities to other animals.

**Figure 1:**
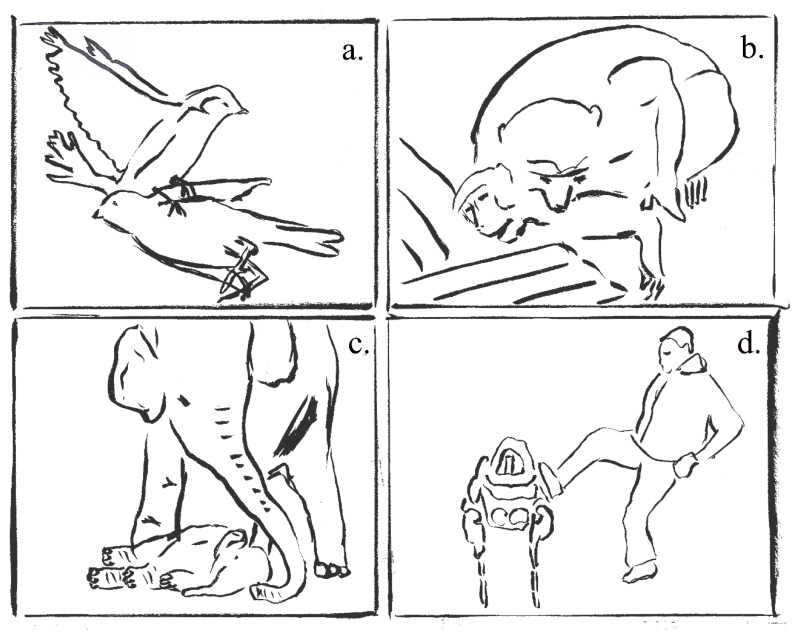
Drawings of the different epimeletic behaviours of (**a**) a sparrow, (**b**) a rhesus macaque, (**c**) an elephant towards an unconscious conspecific. (**d**) An engineer kicking a dog-robot. Picture credit: Cédric Sueur.

In this study, participants answered an online questionnaire about videos showing animal behaviours. The videos are live-action and Figure 1a–d. provide drawn representations of them. Participants observed the videos and then answered the questionnaire. The first three videos showed an individual (a sparrow, an elephant and a macaque, respectively) displaying behaviours towards an inanimate conspecific who suddenly regained consciousness at the end of the footage. These behaviours are interpreted as *epimeletic*, meaning relating to altruistic behaviour towards an injured animal, mostly described in dolphins [81,82,83,84,85,86,87]. Epimeletic behaviours in animals are maybe the most adequate behaviours to study anthropomorphism as these behaviours are very rarely observed and impossible to test (we will not deliberately injure or kill an individual to assess whether its congeners will save it), and finally, researchers have very little knowledge of the underlying beliefs and mental states of death or fear of death in animals [88,89,90,91]. So, only experts on this topic can answer the questions and answer correctly, while the majority of people display anthropomorphism. Scientists advocate a mindful approach to anthropomorphism in these domains (responses to death and prosocial behaviours) [92].

A fourth video showed a robot dog being kicked by an engineer to demonstrate its stability. After each video, questions were asked to score the degree of anthropomorphism shown by participants from mentaphobia (no attribution whatever the species) to full anthropomorphism, and thus measure how accurately the participants evaluated the behaviour in comparison to biological reality (current scientific knowledge of each species). These questions were designed to measure how far participants attribute intentions, beliefs or mental states to non-human animals and robots. We analysed the distributions of these two scores (Mental State attribution Score and Biological Reality Score) globally and per species, then compared them to several socio-demographic variables provided by the participants in the questionnaire. We expect women and participants who have pets or work with animals to show more anthropomorphism than men or people who do not interact with animals, as shown in previous studies [17,93,94,95,96,97]. We also expect that education will lead to less anthropomorphism or to reflective anthropomorphism. Participants are also expected to show more anthropomorphism for species that are phylogenetically close to humans [98].

## 2. Materials and Methods

### 2.1. Research Ethics

All data were anonymous, and participants were given sequential numerical identities according to the date and time they answered the questionnaire. Participants had the possibility to be informed about the study and its results by contacting us via the email address provided in the questionnaire. We followed the ethical guidelines of our institution (CNRS-IPHC, Strasbourg, France).

### 2.2. Design of the Questionnaire

We built a straightforward questionnaire on Google Forms and sent it out via two social media platforms (Facebook and Twitter) and mailing lists, asking people to widely disseminate our survey through their networks. The initial list of recipients were friends but not colleagues of the authors in order to avoid obtaining a set of participants who all work with animals. These friends were asked to forward the link to their friends in order to increase the likelihood of having a pool of participants who were representative of the French population.

The questionnaire was in French and was sent to French citizens. It was composed of questions about the socio-demographic variables of participants, their possible perception of intentionality and awareness of animals displaying specific behaviours on the videos, and the participants’ feelings when watching the robot dog being kicked by the engineer.

Four videos were shown (three concerning animals and one concerning a robot dog), followed by questions.

The first three videos showed the behaviour of an individual towards an unconscious conspecific in (a) a sparrow (*Passer domesticus*; video available here https://youtu.be/wphd1HjT6mg), (b) a rhesus macaque (*Macaca mulatta*; video available here https://youtu.be/_hivJjO2btA) and (c) an elephant (*Elephas maximus*; video available here https://youtu.be/_mKfJuEJk8E) helping a conspecific (Figure 1). Videos were presented in random order to ensure that the answer given for one species did not influence the answers given for the two others. A preliminary study showed that the spoken commentary on the elephant video did not have any effect on the participants’ feelings (i.e., they answered that they did not notice the voice).

The same three questions were asked for each animal species. These questions are:

(Q1) Do you think that this individual had the intention to save its inanimate conspecific by acting like this? Possible answers: ‘Yes’, ‘No’, ‘Maybe’.
(Q2) Do you think that this individual was aware of the risk of imminent death of its conspecific? Possible answers: ‘Yes’, ‘No’, ‘Maybe’.
(Q3) Do you think that individuals of this species are aware of what death is? Possible answers: ‘Yes’, ‘No’, ‘Maybe’.

The terms ‘intention’ and ‘aware’ were deliberately used in these questions to acknowledge the participants’ possible belief that the animals in each video may have been acting on an emotional response or other mental states that a human may experience when seeing another person who is unresponsive/unconscious. These questions were designed to measure the degree of anthropomorphism of participants.

After these three videos, participants watched a fourth video. This video does not show epimeletic behaviour but can cause anthropomorphism. This video (available here: https://youtu.be/4NzcB6TMzjw) showed a four-legged robot that looks like a dog. At two points in the video (at 24” and 30”), an engineer kicks the robot dog to demonstrate its stability (but see also other videos by Boston Dynamics [99,100]). After watching the video, the participants had to answer two questions:

(Q4) What do you feel when the man kicks the robot and that the latter is destabilised? Possible answers (non-exclusive): ‘Surprise’, ‘Amusement’, ‘Anger’, ‘Sadness’, ‘Nothing’.
(Q5) Do you think your feeling is justified? Possible answers: ‘Yes’, ‘No’, ‘Maybe’.

If participants reacted with an emotion such as ‘anger’ or ‘sadness’ when seeing the dog being kicked, it is because they showed anthropomorphism. The answer ‘nothing’ was evidence of reasoning. Indeed, attributing feelings to a robot that has no sensors permitting it to ‘feel’ is a matter of anthropomorphism. The emotional response was due to the target (the robot) of the violent act instead of a response to the violent act itself (kicking) as comments of people watching the video indicate their feeling about the robot (e.g., ‘sad for robot dog—humanity for all’, ‘Did anyone else feel bad when they kicked it’, ‘That robot is going to remember this one day’, ‘I just contacted PETA. They should not of kick the thing. That is just cruel.’).

The socio-demographic questions asked to participants concerned:

(1) Their age: possible answers ‘under 20 yo’, ‘20 to 35 yo’, ‘36 to 50 yo’, ‘51 to 65 yo’, ‘older than 65 yo’ (*yo* is an abbreviation for ‘years old’);
(2) Their gender: ‘woman’, ‘man’, ‘not specified’;
(3) Their academic levels: possible answers ‘no qualifications’, ‘Brevet (GCSEs)’, ‘Baccalauréat (A levels)’, ‘Bachelor’s degree’, ‘Master’s degree’, ‘PhD’;
(4) Whether their profession was linked to animals; possible answers ‘Yes’, ‘No’;
(5) Whether they had at least one pet at home; possible answers ‘Yes’, ‘No’;
(6) Whether they belonged to an Animal Protection Association: possible answers ‘Yes’, ‘No’.

### 2.3. Data Analysing

In the first step of our analyses, we identified which factors may determine the level of anthropomorphism of participants. We calculated an Attribution of Mental States Score (AS) and attributed scores to the different questions (from Q1 to Q4). For Q1 to Q3 (repeated for each species), we gave the following scores: 0 if the participant answered ‘No’; 0.5 if the participant answered ‘Maybe’, 1 if s/he answered ‘Yes’. For Q4, a score of 0 was attributed when the participant answered ‘Nothing’, 0.5 for ‘Surprise’ and ‘Amusement’ (secondary emotions), and 1 for ‘Anger’ and ‘Sadness’, which are part of the primary emotions. As several answers were possible for Q4, we retained only the highest score. The three questions for each of the three species and one question for the robot made a maximum possible score of 10. We therefore obtained an AS score that ranged from 0 for participants demonstrating mentaphobia to 10 for those demonstrating full anthropomorphism (i.e., participants considered animals and robots to show the same mental states as humans, whatever their personal knowledge or expertise concerning these entities). Some non-human animal species have been scientifically identified as sentient and as possessing advanced cognitive skills analogous to human behaviours. One may wonder if we can still talk about anthropomorphism when interpreting the behaviours of these species. In this study, the idea of anthropomorphism is still relevant for two reasons: first, the lack of expertise in animal behaviour for most of the participants will lead them to hypothesise, and second, even if the behaviours and the underlying mental states are analogous to those observed in humans, the subjective nature of consciousness means that they are not similar [32,101,102]. This anthropomorphism with absence of knowledge is called ‘anthropomorphism by omission’ [39]. Conversely, when people attribute mental states to animals because they already have scientific knowledge of the animal’s ability to show them, the anthropomorphism is called ‘reflective’ or ‘critical’ [9,38]. Moreover, it is both valid and viable to talk about anthropomorphism here, particularly because we measure human reactions and human characteristics.

In a second step, we identified which factors determine how close humans are to the biological reality of the animal behaviours observed in the three videos. We calculated a Biological Reality Score (BRS). Biological reality is defined here as what we currently know from the literature and from the scientific observations of animal mental states (consciousness, intentionality). To define a benchmark, we asked international experts in primatology and animal behaviour (N = 14, named in the Acknowledgments section) to answer the same questions as the participants (from Q1 to Q3). We then compared the answers of the two communities. Even if we cannot be one hundred percent certain that the responses of experts reflect biological reality, meaning the true and correct mental states of animals, they all provided similar responses, thus indicating a high probability that they were correct [103,104]. Experts answered ‘No’ for all questions in birds, mainly answered ‘No’ for the monkey (50% answered ‘No’ for Q1, 86% ‘No’ for Q2, and 76% ‘No’ for Q3) and were very likely to attribute intentions to the elephant (0% answered ‘No’ for Q1, 0% ‘No’ for Q2, and 50% ‘No’ for Q3). Based on these responses, we considered the correct answers to be:

- ‘No’ for the three questions about the bird,
- ‘Maybe’ for Q1, and ‘No’ for Q2 and Q3 about the monkey,
- ‘Yes’ for Q1 and Q2 but ‘Maybe’ for Q3 for the elephant.

A score of 1 was attributed when a participant gave the same answer as the experts, 0.5 when s/he answered ‘maybe’, and 0 when the participant gave a different answer to that of the experts. The BSR score ranges from 0 (far from the expert opinions) to 10 (identical to expert opinions), with 10 defined as participants demonstrating a knowledge of biological reality.

We used the Chi-square test to compare different absolute frequencies. The Kolmogorov-Smirnov test and a Spearman rank correlation test were used to compare the distributions of the two scores (AS and BRS). GLM, with a quasi-Poisson law, tested the influence of the six socio-demographic variables (age, gender, academic level, profession, pet ownership and animal right membership) and the influence of the order of questions on both scores. The significance level was set at 0.05. Statistical analyses were performed with the statistical software R 3.5.0 (R core Team, R Foundation for Statistical Computing, Vienna, Austria).

## 3. Results

### 3.1. Participants

**Figure 2:**
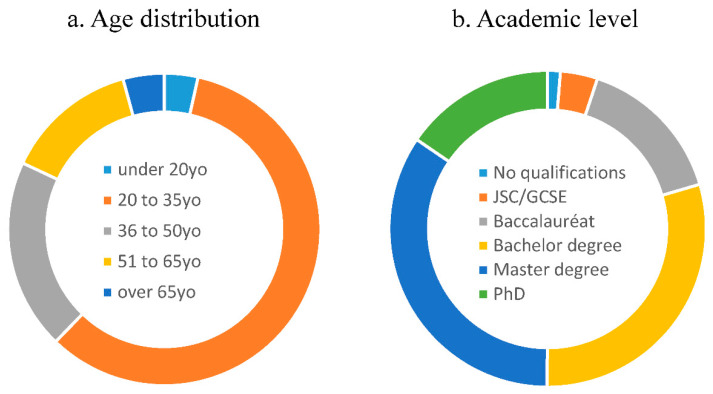
Age and academic levels distribution of the 2160 participants in the study. JSC is junior school certificate (GSCE or Brevet). The Baccalauréat is the equivalent of ‘A’ levels’.

The questionnaire was completed in its entirety by 2160 respondents. Age distribution and academic level distribution are provided in Figure 2. Sample group composition was 72.5% women, and 26.8% men (0.7% did not define their gender). Forty-two point five percent of participants worked in an animal-related profession. Seventy-six point eight percent had a pet at home, and 26.5% were members of an animal protection association. Ratio imbalance (gender and academic level) is offset by sample size and was taken into account in statistical analysis.

### 3.2. Distribution and Comparisons of Scores

**Figure 3:**
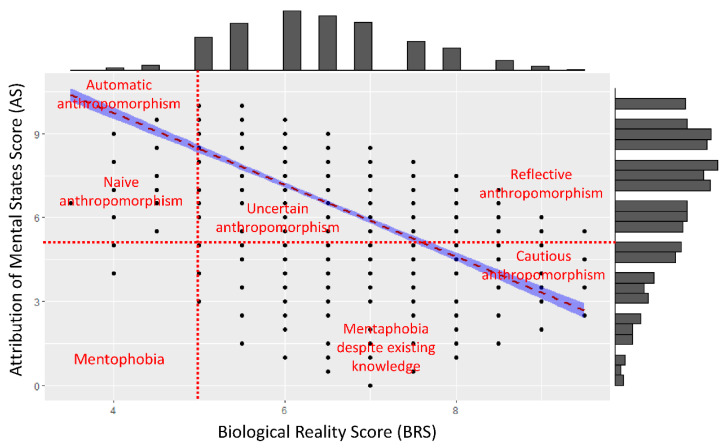
Frequency distribution (histograms) for the Attribution of Mental States Score (AS_TOTAL, y) and the Biological Reality Score (BRS_TOTAL, x). The red dashed line indicates the regression line between the two distributions, with the confidence interval in blue. According to this regression line and the middle score (i.e., 5) for AS and BRS, we divided the figure into different parts indicating anthropomorphism: from mentaphobia to automatic anthropomorphism to reflective (or critical) anthropomorphism.

The distribution of the Attribution of Mental States Score (AS) and the Biological Reality Score (BRS) are shown in Figure 3. The two distributions are different (W = 1,311,700, *p* < 0.0001) but correlated (*p* < 0.0001, r = −0.61). The mean AS is 6.6 ± 2.3 (min = 0; max = 10; median = 7) whilst the mean BRS is 5.1 ± 1.4 (min = 2; max = 9; median = 5). The AS average score is 6.6/10, i.e., above a random mean (5/10, Wilcoxon paired rank test, V= 1,730,700, *p* < 0.0001), and shows that very few participants display mentaphobia (16 out of 2161, i.e., 0.7%) whilst the strongest frequencies are among the highest scores. However, the distribution of the BRS score does not differ from a random distribution (Wilcoxon paired rank test, V = 923,280, *p* = 0.08).

**Figure 4:**
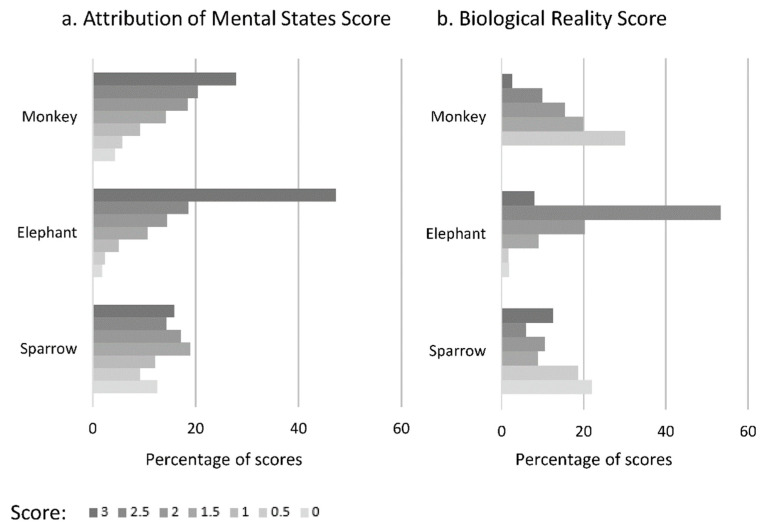
Distribution for each animal species of (**a**) Attribution of Mental States Score and (**b**) Biological Reality Score as percentage of answers.

The distribution of AS and of BRS for each species are provided in Figure 4a,b. The scores are not distributed in the same way according to the species for both AS (chi^2^ = 756, df = 2, *p* < 0.0001; *p*-value for all post-hoc tests < 0.05) and BRS (chi^2^ = 1582, df = 2, *p* < 0.0001; *p*-value for all post-hoc tests < 0.05). 

As regards the Attribution of Mental States Score, the lowest mean but the highest variance concerns the sparrow (1.62 ± 0.93), followed by the monkey (2.05 ± 0.75) and the elephant (2.39 ± 1.57). The lowest Biological Reality Score but with the highest variance concerns the sparrow (1.17 ± 1.00), followed by the macaque (1.30 ± 0.51) and the elephant (2.18 ± 0.37).

**Figure 5:**
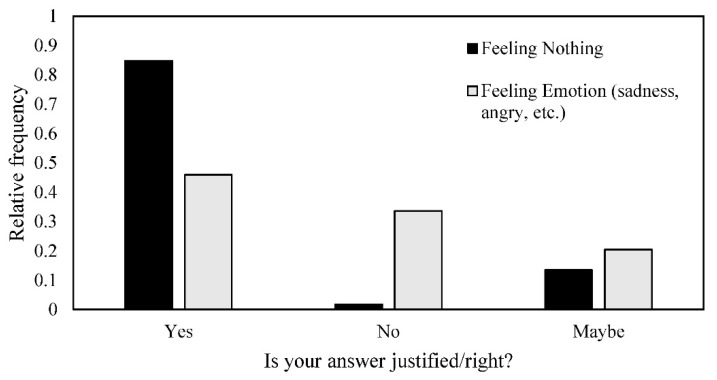
Relative frequency of answers Yes/No/Maybe to the question ‘Do you think that this feeling is justified?’ for participants who said they felt nothing or felt an emotion (sadness, surprise, angry) when the robot was kicked by an engineer.

As regards the robot dog, 645 (29.8%) of the 2160 participants answered that they did not feel any emotion when it was kicked, 1233 (57.1%) answered that they experienced one emotion, 256 (11.8%) answered that they experienced two feelings, 26 (1.3%) answered that they felt three proposed feelings. Among the different feelings indicated by participants, the most common is sadness (36.1%), followed by surprise (34.8%), and anger (22.7%). Only 6.3% of participants answered ‘amusement’. One thousand two hundred and forty-two (57.5%) participants considered that their feeling was justified, 397 (18.4%) answered ‘Maybe’ and 521 (24.1%) claimed that their feeling was not justified. However, this distribution was different when we compared the participants who answered that they felt nothing and those who answered that they felt something (regardless of the emotion felt, Figure 5; chi^2^ = 321, df = 5, *p* < 0.0001).

### 3.3. Socio-Demographic Factors Affecting Scores

The factors used in the model and their statistical effect are shown in Table 1 for the Attribution of Mental States Score and in Table 2 for the Biological Reality Score. Effect size is indicated by the *t*-value. Usually, an absolute *t*-value higher than 1.9 indicates a significant effect (*p* < 0.05). The higher the absolute *t*-value, the stronger the effect. Age influences the Attribution of Mental States Score. Young participants (under 20 yo) have a lower score than those over 65 yo. Other age pair comparisons are not significant (*t*-value > 1.108, *p* > 0.268). However, no age effect is found on the Biological Reality Score. Gender influenced both scores, with men displaying less anthropomorphism and being closer to biological reality than women. As regards the academic levels of the participants, only participants with a PhD appear to be different to participants with no diploma. Other academic pair comparisons are not significant (*t*-value > 1.801, *p* > 0.071). Participants with PhDs demonstrate less anthropomorphism and obtain higher biological reality scores.

Participants whose profession involved animals obtained a lower Attribution of Mental States Score and a higher Biological Reality Score. The same double effect was observed for participants who had a pet at home. However, the opposite effect was observed for participants who were members of an animal protection association: they displayed more anthropomorphism than other participants and obtained a lower biological reality score.

The order of questions also has an impact on both scores: when questions about the monkey were shown first and those about the birds last, participants obtained a lower score for anthropomorphism and a higher Biological Reality Score than when questions were asked in the opposite order. When looking at each species, the Attribution of Mental States Score is higher, whatever the species, when the video of the sparrow is shown first, but the highest difference between the two conditions of question order is observed for the monkey (sparrow video shown first: sparrow = 1.69 ± 0.89, elephant = 2.46 ± 0.71, macaque = 2.26 ± 0.81; monkey video shown first: macaque = 1.88 ± 0.87, elephant = 2.33 ± 0.77, sparrow = 1.59 ± 1.01).

## 4. Discussion

This study sought to understand the anthropomorphism process by showing participants three videos of animals in difficulty and one video of a kicked robot. The events we showed (epimeletic behaviours in animals and kick of a robot) produced a negative correlation (about 61%) between the Attribution of Mental States Score (AS) and the biological reality one (BRS). Moreover, there is wide AS heterogeneity for any given BRS (see Figure 3). A positive correlation between AS and BRS indicates a correct statement from participants (they answer correctly with more reflective anthropomorphism), while a negative correlation shows an incorrect statement: they answer incorrectly using mostly automatic anthropomorphism (but see Figure 3 for the different mechanisms proposed). Indeed, the heterogeneity (i.e., the remaining 39% of variance) and distribution of the AS scores proved that all levels of anthropomorphism are shown, from mentaphobia (not giving attributes to animals even if it is appropriate) to full anthropomorphism. Our results allowed us to identify other types of anthropomorphism: naïve anthropomorphism (i.e., the participant displayed anthropomorphism due to a lack of knowledge but did not do so systematically, similar to anthropomorphism by omission), uncertain anthropomorphism (i.e., the participant displayed anthropomorphism despite some personal knowledge but not giving it systematically) and cautious anthropomorphism (i.e., the participant displayed anthropomorphism but less than would be expected on the basis of current scientific knowledge, because participants either do not have this expertise, or know about recent studies but remain sceptical about them). Very few participants exclusively showed mentaphobia or reflective (‘critical’) anthropomorphism. This heterogeneity in the way humans attribute mental states to animals depends on several factors such as the animal species considered, the behaviours displayed by the animals but also factors that are based on human experience (personal but also general, along with current scientific knowledge) and personality (i.e., mainly the degree of empathy of people in this study [67,68,105]).

Different cognitive processes (i.e., agency detection, social cognition, motor-matching mechanisms, empathy for pain, mental representations, inductive and causal reasoning) are involved in anthropomorphism [9]. These mechanisms mostly enable individuals to deal with objects in everyday life, including animate entities such as animals (e.g., finding resources by observing other animal species, interpreting interspecific and intraspecific threats, or avoiding predation). The posterior superior temporal sulcus [69] and mirror neurons [70] are used to analyse social information concerning the same species (*Homo sapiens*, in our case) but also to react to other species [71], as well as the movements or facial expressions of robots [72,73]. Thus, humans inevitably and unconsciously anthropomorphise, as confirmed by our score. The negative correlation with the BRS shows the relatively systematic anthropomorphism of participants. However, the scores they attributed to animals differed according to the species shown in the video. The sparrow video produced the lowest AS value of the videos, but these results also showed the highest level of variance (meaning that the answers differed more for this species than for the others). Participants attributed more mental states and cognitive processes to the macaque and the elephant, and their responses were more homogenous. Miralles and colleagues [98] showed that empathy and compassion toward other species (animal and non-animal) decrease with evolutionary divergence time. Urquiza-Haas and Kotrschal [9] argued that as the phylogenetic distance increases between a human and the species it observes, anthropomorphism reduces or becomes reflective rather than automatic (i.e., visceral). This reflective attribution of intentions is mirrored in the heterogeneity (variance) of the answers (as for the sparrow) [106]. Following this logic, a low variance, meaning a high level of agreement, should indicate causal reasoning whilst an automatic or implicit anthropomorphism would be expected when heterogeneity is observed in the attribution of mental states. In addition to phylogenetic distance, the sharing of common morphological and behavioural traits by the observer and the subject may also result in a stronger likelihood that the former will anthropomorphise. This explains why the highest difference between AS and BRS is observed for the macaque video, where the monkey appears to massage its unconscious partner in order to reanimate him. However, none of the 13 experts we questioned were convinced that the monkey intended to reanimate his conspecific or about the macaque mental representation behind this behaviour. Although no systematic studies to date have proved that sparrows or rhesus macaques are aware of death, there is some evidence that has led researchers to think that elephants probably perceive loss of life [88,91,107]. So, to summarise, whilst participants showed more automatic anthropomorphism when watching the sparrow, thus leading to a higher variance, anthropomorphism was more reflective for the macaques and the elephant.

Participants showed more automatic anthropomorphism when they watched the video of the robot, which shared common morphological and behavioural traits with dogs (four legs and same movement). This is confirmed by the high number of sadness and surprise answers we obtained. However, it is very striking that people were not sure about the validity of their feelings: only 45.9% of certainty was observed when people feel an emotion versus 84.8% for participants saying that they felt nothing. This shows that participants reacted and felt emotions but then doubted the validity of their feelings (is it right to feel this emotion?). Gazzola et al. [108] already showed this automatic process in humans observing robots. The way humans reacted here depends not only on the animal species but also on their movements and actions, as explained above [109,110].

When the anthropomorphism becomes more automatic, more heterogeneity is observed in the attribution of mental states. But what explains this variance? As we briefly suggested in the introduction, many of the factors examined in this study may play a role in the way we perceive animals’ minds. The first factor involves the cognitive biases and automatic reactions discussed above, namely the order of questions. The first video of the animals and the actions had an influence on the participants’ AS: when the video of the bird was shown first, the global score and the score for each species was higher. The greatest difference between the two order conditions was observed for the rhesus macaques: the AS increased by 0.5 point when participants watched this video last. They were influenced by the first two videos, and this increased the score for the final video. This bias is well known in psychology. It is comparable to the anchoring bias and is found in humans [60] and in other species [58]. This bias may lead to suboptimal decisions [111] because individuals are influenced by previous actions in their actual decision.

Age also affects AS (but not BRS), with older participants being less likely to attribute mental states to animals. However, this is not explained by their experience and knowledge (as the BRS does not increase with age). This result might be explained by generational differences in the perception of animals (and robots) through media such as televisions or books. Animals or inanimate objects such as robots (but also trains or cars) are increasingly anthropomorphised in cartoons and comics, and the language or the images used to describe animals or objects has an effect on children’s tendency to attribute human traits to the latter [112,113]. A number of studies have shown different results describing the positive or negative impact of this everyday anthropomorphism on animal conservation and welfare [10,114]. For instance, anthropomorphic selection (e.g., the genetic selection by humans of morphological traits such as eyes, nose or ears that make animals appear more human) is one of the more severe welfare problems affecting pets, particularly dogs [115]. Alternatively, the use of *humanizing* language to emphasise the human qualities of dogs increases beneficial action for them, and could thus be used to improve animal welfare [116].

Gender also affects the attribution of mental states, with women demonstrating more anthropomorphism. The difference between men and women has been well described for cognitive processes such as decision-making [117], risk taking [118], sociality [119] and empathy [120,121,122]. Oxytocin, a hormone involved in offspring recognition, sociality and empathy, has been directly linked to attitudes towards animals [105]. Gender is one of the strongest predictors of caring about animals, with women being more protective of animals in different situations [67,123,124,125,126]. This general gender effect might be due to the evolutionary history of animal females to protect their offspring [127,128] and the link between social cognition and morphism [9].

Interestingly, academic level does not have a great impact on the attribution of mental states: only PhD participants obtained lower AS and higher BRS values than participants with no qualifications. This may confirm the impact of social media we described in a previous paragraph: animals are now commonly described in the media, with specific magazines such as National Geographic or BBC Earth [129,130,131], and people may have a good general knowledge about animals. Only people with a PhD who are experts in animal behaviour or similar domains are more careful about their interpretations. Similarly, people who work with animals or have at least one pet at home demonstrate less anthropomorphism and appeared closer to reality in their mental state attributions than those who do not. This might mean that being with animals on an almost daily basis makes people more cautious in their interpretations when observing their behaviour [132]. People who work with animals and have substantial experience in jobs in which they are emotionally involved may show less naïve anthropomorphism towards animals. Indeed, people working in the domain of animal physiology, such as vets, take emotional distances from the animals they encounter [124,133] in the same way as surgeons who operate on humans.

An interesting finding of this study is that people who have at least one pet at home demonstrate less anthropomorphism; this is surprising as a pet is usually considered to be a member of the family [134]. More interestingly, Albert and Bulcroft [135] showed that a person whose status changes to single, divorced or remarried displays more anthropomorphism thereafter, projecting human qualities such as loyalty to their pet. This trait-based behaviour can be related to sociality motivation, i.e., the need to feel socially connected with others [97]. Moreover, some pet owners celebrate the birthday of their animals [96]. Our result contradicts the literature. This may be explained by the fact that the animals shown in the current videos are not pets but wild animals. Participants do not attribute the same mental states or demonstrate the same empathy to these two categories of animals, pets and wildlife. The most striking example of this cognitive dissonance is the attitude of cat owners towards the impact of their pet on biodiversity [136,137,138,139,140]. Whilst they care about the welfare of their cat and of cats in general, they show less concern for the animals that are injured or killed by their cat [141] and by the consequent loss of biodiversity [142,143]. In this moral schizophrenia [144,145], concern for biodiversity and other animals can never exceed the pet owners’ compassion for cats [146]. The relationship owners develop with their pets increases levels of oxytocin, which in turn directly influences on empathy and anthropomorphism. However, the latter is selective and is displayed towards the owner’s own pet rather than other animals [94,105,147].

Conversely, we found that animal protection association members demonstrated strong anthropomorphism and were much more distant from biological reality than non-members. Like vegetarians, members of these associations are expected to show higher levels of anthropomorphism and empathy. Anthropomorphism and empathy are different but are strongly linked, as empathy is one of the cognitive processes explaining anthropomorphism [9]. The empathy-related areas in vegetarians’ brains are more active than those of omnivores [74,148]. As these individuals are usually more empathic (towards humans, but see for instance [149,150]) and have higher perspective-taking abilities (i.e., adopting someone else’s perspective), they are systematically more empathic towards animals and therefore more likely to become a member of an animal protection association [131].

The large set of participants obtained for this study confirmed our expectations about factors influencing anthropomorphism. However, we cannot exclude bias in this study, as the participants who decided to answer the questions were already interested in the topic. For instance, the sample showed an over-representation of women as well as of people belonging to an animal protection association or who had a pet. Whilst this bias does not change our statistics about socio-demographic factors influencing the scores (Section 3.3.), it could influence the distribution of scores, both globally and per species (Section 3.2.). Moreover, the questionnaire was sent exclusively to French citizens, and we know that the cultures of humans influence their position regarding animals [19,20,66]. It is therefore important to continue this study on other datasets. We also identified anthropomorphism as the main mechanism underlying the answers of participants, but anthropomorphism may be just one of a number of possible explanations. For instance, in a study of human-robot interaction, five key concepts were identified: anthropomorphism, animacy, likeability, perceived intelligence, and perceived safety [151]. It is difficult to evaluate how far these other concepts occur independently of anthropomorphism and further research is needed on this subject.

This study determined several factors influencing how much humans anthropomorphise: some of these factors depend on the animal species or behaviour and others are linked to human characteristics. Understanding the potential usefulness of these factors can lead to better relationships with animals and encourage human-robot interactions [152,153]. Last but not least, such anthropomorphism can lead to an increase of human empathy and sociality, thus inevitably increasing our humanity (universal virtue as defined in [154]). These variables could also be used as a tool to solve biodiversity conservation problems as proposed for charismatic and uncharismatic species in both vertebrates and invertebrates [10,11,155,156,157,158]. Indeed, public attention and the interest humans show towards endangered species is a crucial prerequisite for effective conservation programs [159]. Notably, more efficient communication is required about the threat of biological invasions and climate change on some species, using the symbols [10] that attract, affect and alert the public. Human emotions play an important role in animal protection and wildlife conservation [160]. Animal ethics is a fundamental question for human beings—not simply because it concerns animals, but because it brings humans back to their original roots. The way we behave towards animals must be a tool to protect them at the individual and species level, and thus also protect ourselves [161,162].

## Figures and Tables

**Table 1:** Statistical values for the GLzM with the Anthropomorphism Score as response variable. For Age, indicated factors are compared with Age [under 20 yo]. For Gender, ‘man’ is compared to ‘woman’. For academic levels, factors are compared to [Without diploma]. For the remaining questions [Yes] is compared to [No]. For the estimate and the *t*-value, a positive value indicates a positive influence of the factor; a negative value indicates a negative influence. Significance: * < 0.05, ** < 0.01, *** < 0.001.

Factors	Estimate	Std. Error	*t*-Value	Pr(>|t|)	Significance
(Intercept)	2.136	0.076	28.143	<0.0001	***
Age [20 to 35 yo]	−0.046	0.040	−1.147	0.252	
Age [36 to 50 yo]	−0.091	0.042	−2.19	0.029	*
Age [51 to 65 yo]	−0.095	0.043	−2.213	0.027	*
Age [over 65 yo]	−0.118	0.052	−2.255	0.024	*
Gender [Man]	−0.064	0.017	−3.708	0.0002	***
Academic Level [Brevet]	0.072	0.072	1.006	0.315	
Academic Level [Baccalauréat]	−0.025	0.065	−0.377	0.706	
Academic Level [Bachelor degree]	−0.015	0.064	−0.236	0.814	
Academic Level [Master degree]	−0.048	0.064	−0.755	0.450	
Academic Level [PhD]	−0.168	0.066	−2.551	0.011	*
Profession involving animals [Yes]	−0.162	0.016	−10.113	<0.0001	***
Has at least one pet at home [Yes]	−0.037	0.018	−2.05	0.041	*
Animal Protection Association membership [Yes]	0.075	0.017	4.507	<0.0001	***
Order of questions (monkey or sparrow first)	−0.089	0.015	−5.973	<0.0001	***

**Table 2:** Statistical values for the GLM with the Biological Reality Score as response variable. For Age, indicated factors are compared with Age [less than 20 yo]. For Gender, ‘man’ is compared to ‘woman’. For academic level, factors are compared to [Without diploma]. For the remaining questions [Yes] is compared to [No]. For the estimate and the *t*-value, a positive value indicates a positive influence of the factor; a negative value indicates a negative influence. Significance: * < 0.05, ** < 0.01, *** < 0.001.

Factors	Estimate	Std. Error	*t*-Value	Pr(>|t|)	Significance
(Intercept)	1.431	0.064	22.29	<0.0001	***
Age [20 to 35 yo]	0.001	0.033	0.04	0.968	
Age [36 to 50 yo]	0.034	0.035	0.981	0.327	
Age [51 to 65 yo]	0.042	0.036	1.184	0.236	
Age [over 65 yo]	0.073	0.043	1.707	0.088	
Gender [Man]	0.036	0.013	2.694	0.007	**
Academic Level [Brevet]	0.001	0.061	0.014	0.989	
Academic Level [Baccalauréat]	0.077	0.055	1.397	0.163	
Academic Level [Bachelor degree]	0.057	0.054	1.052	0.293	
Academic Level [Master degree]	0.082	0.054	1.507	0.132	
Academic Level [PhD]	0.116	0.055	2.112	0.035	*
Profession involves animals [Yes]	0.135	0.012	10.972	<0.0001	***
Has at least one pet at home [Yes]	0.029	0.014	1.981	0.048	*
Animal Protection Association membership [Yes]	−0.049	0.013	−3.677	0.0002	***
Order of questions (monkey or sparrow first)	0.047	0.012	4.012	<0.0001	***
